# Concomitant Radiotherapy and Chemotherapy for High-Risk Nonmelanoma Skin Carcinomas of the Head and Neck

**DOI:** 10.1155/2011/464829

**Published:** 2011-09-25

**Authors:** Smith Apisarnthanarax, Nirav Dhruva, Farhad Ardeshirpour, Joel E. Tepper, Carol G. Shores, Julian G. Rosenman, William W. Shockley, Michele C. Hayward, D. Neil Hayes

**Affiliations:** ^1^Department of Radiation Oncology, University of Pennsylvania, Abramson Cancer Center, Philadelphia, PA 19104, USA; ^2^Department of Medical Oncology, University of North Carolina Hospitals, Lineberger Comprehensive Cancer Center, Chapel Hill, NC 27599, USA; ^3^Department of Head and Neck Surgery, University of North Carolina Hospitals, Lineberger Comprehensive Cancer Center, Chapel Hill, NC 27599, USA; ^4^Department of Radiation Oncology, University of North Carolina Hospitals, Lineberger Comprehensive Cancer Center, Chapel Hill, NC 27599, USA

## Abstract

*Background*. To report on the use and feasibility of a multimodality approach using concomitant radiotherapy and chemotherapy in patients with high-risk nonmelanoma skin carcinoma (NMSC) of the head and neck. *Methods*. Records of patients with NMSC of the head and neck who received concomitant CRT at the University of North Carolina between 2001 and 2007 were reviewed. *Results*. Fifteen identified patients had at least one of the following high-risk factors: T4 disease (93%), unresectability (60%), regional nodal involvement (40%), and/or recurrence (47%). Ten patients were treated in the definitive setting and five in the postoperative setting. Platinum based chemotherapy was given in 14 (93%) patients. Ten of fifteen (67%) patients completed all planned chemotherapy treatments, and thirteen patients (87%) completed at least 80% of planned chemotherapy. Mild radiation dermatitis occurred in all patients and reached grade 3 in 13% of patients. No patients experienced grade 4 or 5 toxicity. With a median followup of 31 months in surviving patients, the 2-year actuarial locoregional control and relapse-free survival were 79% and 49%, respectively. *Conclusions*. Definitive or postoperative chemoradiotherapy for patients with locally advanced or regionally metastasized NMSC of the head and neck appears feasible with acceptable toxicities and favorable locoregional control.

## 1. Introduction

Nonmelanoma skin carcinoma (NMSC) is the most common malignancy worldwide with an incidence of over 1.3 million in the United States, making it an important global public health issue [1]. NMSCs arise in anatomic areas subject to frequent sun exposure, most commonly in the head and neck [2]. When diagnosed at an early stage, localized squamous and basal cell carcinomas of the skin have high cure rates of greater than 90% with local treatments such as surgical excision, Mohs' chemosurgery, electrocautery, and radiotherapy (RT) [3, 4]. 

However, subsets of these cancers can be biologically and clinically aggressive with a greater propensity for local, regional nodal, and, to a lesser extent, distant metastatic spread [5]. These “high-risk” NMSCs have been reported to be associated with certain adverse prognostic features, including large tumor size, high grade, deep invasion, regional nodal involvement, recurrent disease, perineural invasion, and immunosuppression [6–8]. 

The optimal management of these high-risk tumors is unclear due to the paucity of data. Despite aggressive treatment with surgery and/or RT, locoregional failures represent the first site of recurrence in the majority (70–80%) [9, 10] of these patients and are associated with considerable morbidity and disease-related mortality [3, 11–14]. Optimizing locoregional control, therefore, may significantly improve long-term clinical outcomes. One proposed method of intensifying locoregional treatment is through a multidisciplinary approach of integrating surgery, RT, and chemotherapy in various combinations.

There has been increasing interest in the use of chemotherapy for NMSCs [15]. The addition of chemotherapy for tumor radiosensitization to increase locoregional tumor control is an approach that could potentially improve outcomes in these high-risk patients. The superiority of chemoradiotherapy (CRT) over RT alone has been established for squamous cell carcinomas in many other tumor sites, including mucosal head and neck [16–19], esophageal [20], cervical [21–23], and anal carcinomas [24, 25]. However, the role of adding chemotherapy to RT in high-risk NMSCs has largely been unexplored with evidence limited to isolated case reports [26, 27]. 

The feasibility and toxicities of combining a systemic radiosensitizing agent with RT in this group of patients are unknown, particularly because these patients possess patient and tumor characteristics, such as older age and superficial tumor location, which may confer different toxicities compared to those experienced in patients treated for mucosal tumors of the head and neck. We report our institutional experience on the feasibility, toxicity, and outcomes of treating high-risk NMSCs of the head and neck with concomitant CRT.

## 2. Patients and Methods

Patients with NMSCs of the head and neck treated with concomitant CRT from 2001 to 2007 were identified by reviewing the head and neck database at the University of North Carolina Hospitals (Lineberger Comprehensive Cancer Center, Chapel Hill, NC, USA). Patients with histologies other than squamous or basal cell carcinoma and NMSC of the lip were excluded, since these patients were treated primarily with surgery alone at our institution. Patients were evaluated by a multimodality team consisting of the head and neck surgeon, medical oncologist, and radiation oncologist. For comparison, records of patients with NMSC of the head and neck treated with RT alone were also evaluated from 1990 to 2007. To select patients with high-risk features, patients treated only for cosmetic purposes or personal preference were excluded from the RT-alone patient group. 

 Clinical and histopathologic data were gathered on patient characteristics, treatment delivered, acute and late toxicities, and treatment outcomes. Patients were staged according to the AJCC cancer staging guidelines for carcinoma of the skin, excluding eyelid, vulva, and penis [28]. Toxicities were scored according to the Common Terminology Criteria for Adverse Events (CTCAE), version 3.0, for chemotherapy and to the Radiation Therapy Oncology Group (RTOG) criteria for RT. Locoregional failure was defined as the reappearance of tumor in the original tumor bed or development of cervical or intraparotid node metastases after treatment. Survival outcomes were measured from the time of treatment start. This study was approved by the University of North Carolina Biomedical Institutional Review Board. 

Differences in means were tested by the Welch two-sample *t*-test. Rates of locoregional control were estimated according to the method of cumulative incidence [29], and differences were assessed by the Gray's test [30]. Rates of relapse-free survival and overall survival were estimated according to the Kaplan-Meier method [31], and differences between groups were assessed by log-rank statistic [32]. Hazard ratios were calculated using the Cox proportional hazard method. Differences in proportions were assessed by the 2-sample test for equality of proportions with continuity correction. All *P* values reported are for 2-sided tests. The statistical software package R 2.9.1 was used for all statistical testing except for rendering of the Kaplan-Meier plots which were performed in GraphPad Prism [33].

## 3. Results

### 3.1. Patient Characteristics

Fifteen patients with NMSC of the head and neck treated with concomitant CRT and 30 patients treated with RT-alone were identified. Median followup in surviving patients was 31 months (range, 9–71 months) for CRT patients and 35 months (range, 2–223 months) for RT-alone patients. Nearly all patients (89%) were male and Caucasian (98%) (Table 1). The median age (66 years) in these patients is expected given the typical age at presentation for NMSCs. The majority of patients in both groups had squamous cell histology (73% CRT, 63% RT). Treatment was considered curative in 29 patients and palliative for aggressive local control in 1 patient for RT alone patients. All patients treated with CRT were treated with curative intent. No patient had immunosuppression. 

**Figure 1:**
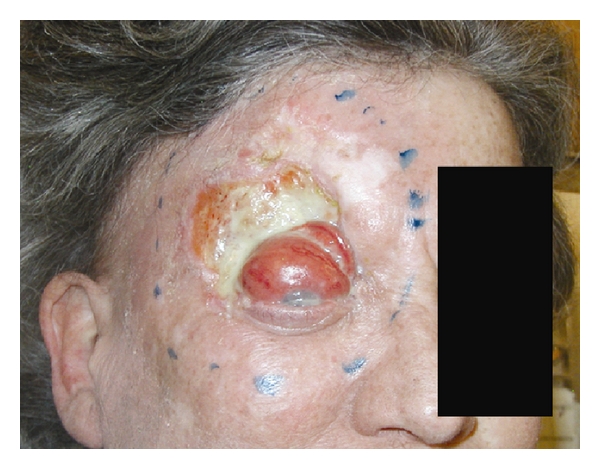
Patient treated with concomitant chemoradiotherapy: 60-year-old white female with an unresectable locally advanced T4 basal carcinoma of the right forehead involving the frontal sinus, lateral orbit, extraocular muscles, and eyelid. She received 70 Gy of radiation given concomitantly with weekly cisplatin 30 mg/m^2^.

Patients treated with CRT had at least one of the following high-risk factors: T4 disease (93%), unresectability (60%), regional nodal involvement (40%), and/or recurrence (47%). Overall, patients in the CRT group possessed more high-risk tumor features compared to RT patients: large tumor size (T stage), nodal involvement, unresectable tumors, bone invasion, soft tissue invasion, extracapsular extension, multiple recurrences, and high-grade status. Of these adverse factors, T stage, unresectability, and bone invasion were statistically different. All patients except 1 in the CRT group had T4 disease (93%) compared to 47% in the RT group. In parallel with the differences in resectability and T stage between the two groups, patients treated with CRT tended to be treated in the definitive treatment setting (67%) compared to the postoperative setting (50%) in RT-treated patients. An example of a patient treated with CRT is shown in Figure 1.

### 3.2. Treatment Delivered

RT was delivered daily for 5 days a week. The median dose of radiation to gross disease was 70 Gy (range: 58 to 75 Gy) in 1.8 to 2 Gy fractions. One patient was planned to receive 70 Gy, but only received 58 Gy due to treatment toxicity. For subclinical (microscopic) disease, the median radiation dose was 50 Gy (range: 46 to 61 Gy) in 1.8 to 2.0 Gy fractions. Photons only were delivered in 3 patients and a combination of photons and electrons in 12 patients. All patients were treated using CT-based three-dimensional treatment planning. Four patients received intensity-modulated radiation therapy (IMRT), primarily for parotid gland sparing. Tissue-equivalent bolus was used in 8 patients to increase the skin surface dose to the tumor. 

In each case, the decision to offer chemotherapy in combination with RT was made at the recommendation of the multidisciplinary head and neck tumor board and in agreement between the treating medical oncologist and radiation oncologist based on overall patient risk. The chemotherapy regimens employed were at the discretion of the treating medical oncologist. A platinum-based regimen was given concomitantly with RT in 14 of 15 (93%) patients. Weekly cisplatin (20–30 mg/m^2^ in 8 patients) or weekly carboplatin (AUC 2 in 2 patients) was used as the sole radiosensitizer in 10 patients, while 3 patients received two agents concomitantly (carboplatin and 5-FU, cisplatin and 5-FU, carboplatin and paclitaxel). One patient received oral therapy with capecitabine, while another received cisplatin 100 mg/m^2^ delivered every 3 weeks.

### 3.3. Treatment Tolerance and Toxicity

In CRT patients, chemotherapy was well tolerated overall with 10 of 15 (67%) patients completing all planned treatments. Thirteen of fifteen (87%) patients completed at least 80% of planned chemotherapy and fourteen of fifteen (93%) completed all planned courses of radiation. Two patients had hematologic toxicity that precluded one cycle of therapy (one patient had a platelet count of 89,000 and one patient had a WBC count of 1.7 with ANC 1.0), while one patient missed one treatment due to nonadherence. The remaining 2 patients missed treatment because of grade 3 mucositis or delirium. Every patient who did not complete all treatments (*n* = 5) received weekly carboplatin or cisplatin. 

During concomitant chemotherapy, no patient experienced grade 4 or 5 acute toxicity (Table 2). The most common acute toxicity was radiation dermatitis, which occurred in all 15 patients and was mild (grade 1 or 2) in nearly all patients (87%). Only 2 patients (13%) experienced grade 3 dermatitis that required radiation treatment breaks of 2 and 6 days, respectively. In one patient, the cheek and maxillary sinus was treated with mixed photons and electrons concomitantly. The other patient was treated with electrons to the primary forehead site and photons to the neck via IMRT. Tissue-equivalent bolus and concomitant weekly carboplatin was used in both of these patients. Six of the other eight patients in which bolus was used developed grade 2 dermatitis. 

Mucositis occurred in 5 patients (33%), reaching grade 3 in only 1 patient (7%). This patient was treated with extensive radiation fields encompassing nearly the entirety of his left face, including the orbit and maxillary sinus, along with weekly cisplatin. Grade 3 keratitis occurred in 2 patients who had tumor directly involving or adjacent to the orbits, which unavoidably received the full prescribed dose (70 Gy and 50.4 Gy, resp.). One patient, aged 84, experienced acute altered mental status changes that required hospitalization for grade 3 delirium. His treatment with concomitant RT and weekly carboplatin was stopped early because of this toxicity.

Grade 3 leukopenia and lymphopenia were observed in 2 and 4 patients, respectively. One patient had a grade 1 creatinine elevation prompting change in regimen from cisplatin 30 mg/m^2^ to carboplatin AUC thrice weekly. Nausea, vomiting, and fatigue were limited to grade 1 or 2 toxicities, and no patient had grade 3 or 4 electrolyte abnormalities.

Serious late toxicities were rare, occurring in only 2 patients. One patient developed grade 3 osteoradionecrosis of the temporal bone, and another patient experienced grade 3 chronic otitis media with associated hearing loss. These toxicities were expected because these normal tissue structures were within the high-risk radiation target volumes and received the full prescribed dose.

### 3.4. Treatment Outcomes

**Figure 2:**
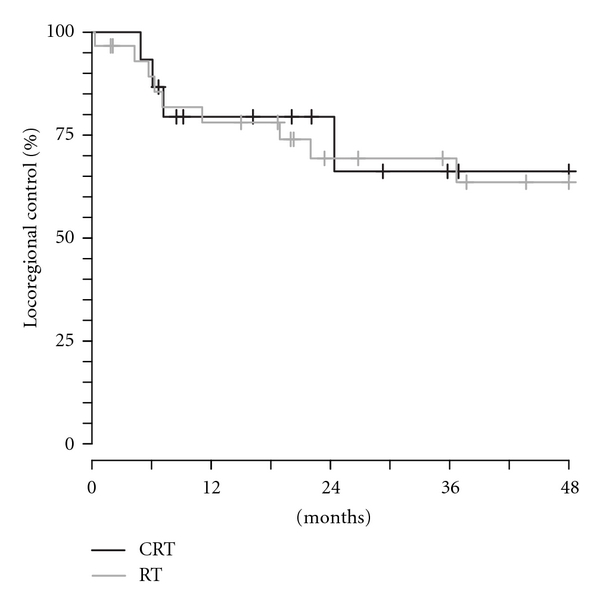
Rates of locoregional control in chemoradiotherapy (CRT) and radiotherapy alone (RT) treated patients.

**Figure 3:**
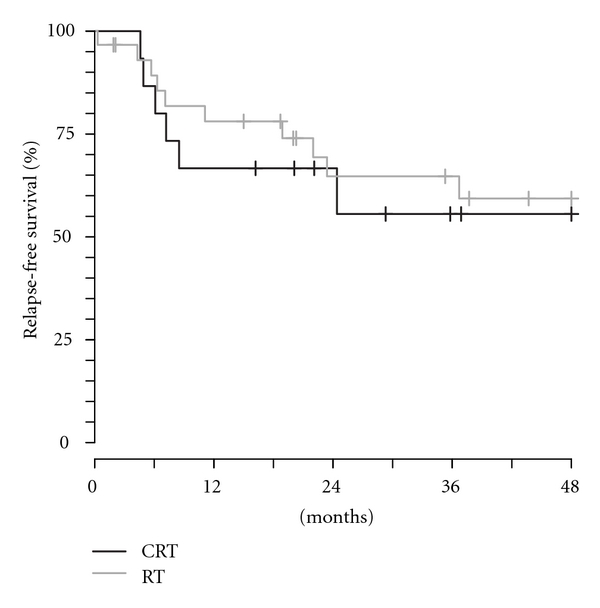
The Kaplan Meier estimates of relapse-free survival. CRT: chemoradiotherapy; RT: radiotherapy.

The estimated 2-year locoregional control rates were 79% for CRT and 69% for RT patients (Figure 2). The median time to recurrence was 7 months, and greater than 85% of the failures occurred within 24 months of treatment. The estimated 2-year relapse-free survival rates for the CRT and RT groups were 49% and 60%, respectively (Figure 3). The estimated 2-year overall survival rates were 65% (CRT) and 86% (RT). Of those who died, 5 patients died with recurrent disease, and 2 patients died without evidence of disease. 

On univariate analysis for relapse-free survival on all patients, there was a trend towards decreased relapse-free survival for soft tissue and nerve invasion, nodal involvement, and positive margins, but these were not statistically significant (Table 3). The only statistically significant factor in the univariate analysis was bone invasion (HR, 5.25; *P* < 0.01).

**Figure 4:**
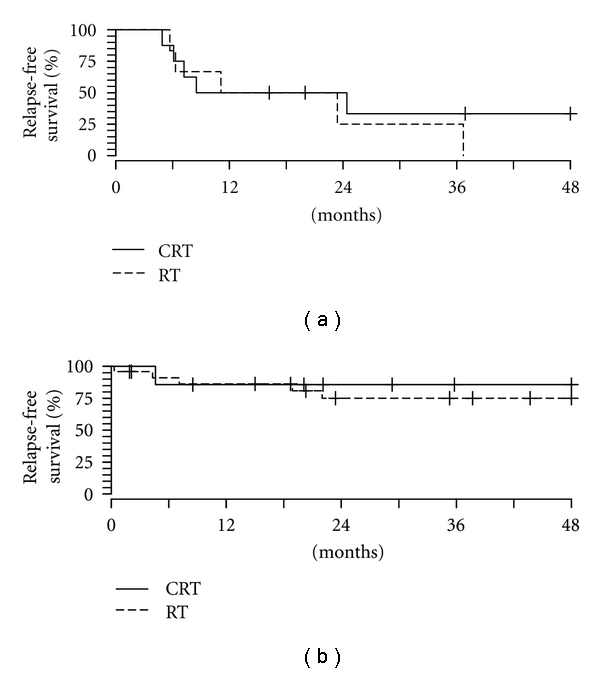
Estimated relapse-free survival in patients with bone invasion (a) and without bone invasion (b) treated with chemoradiotherapy (CRT) and radiotherapy (RT).

On multivariate analysis, both bone invasion (HR, 9.43; CI, 2.8–32; *P* < 0.01) and nodal involvement (HR, 2.60; CI, 1.3–5.1; *P* = 0.01) were highly statistically significantly associated with worse outcome. As previously noted, bone invasion was associated with the decision to add chemotherapy to RT. Therefore, we investigated the potential confounding on estimates of treatment effect by bone invasion status. When controlling for bone invasion and nodal involvement by Cox proportional hazards modeling, we detected a trend toward clinical benefit of combined modality CRT over RT alone for relapse-free survival (HR, 2.72; CI 7.9–9.3; *P* = 0.11). Patients with bone invasion had poor relapse-free survival rates in both treated groups (25% at 3 years), whereas patients without bone invasion had improved outcomes (86% and 68% at 3 years for CRT and RT, resp.) (Figure 4). 

### 3.5. Patterns of Failure

Of the 6 patients who developed recurrences after treatment with CRT, 2 patients developed distant failures (dermal metastasis and lung, bones, adrenals); both patients had advanced nodal disease at diagnosis (Table 4). Four local or regional failures were observed in the CRT-treated patients; three were isolated local failures and one was both local and regional failure.

## 4. Discussion

Although NMSC is common worldwide, the prevalence of the most aggressive forms of the disease is unknown, as most cancer surveillance registries do not track NMSC. We estimate that approximately 2–4% (or 6 patients/year) of all head and neck tumors treated at our institution have a diagnosis of NMSC over the years 2001 to 2007, making this an uncommon, but not rare, disease in our practice. Currently, no clear established role exists for chemotherapy in the definitive or adjuvant treatment of NMSCs, either alone or in combination with RT or surgery. This study is the first report on multiple patients with high-risk NMSCs of the head and neck treated with concomitant RT and chemotherapy. 

Our experience suggests that delivering chemotherapy concomitantly with RT is feasible and generally well tolerated with minimal morbidity as delivered in our patient population. Many of the chemotherapy regimens used in our patients have a history of acceptable tolerability and success for other cancers that employ CRT, most notably mucosal head and neck cancer. Nearly all patients were able to complete the planned therapy: 87% of patients completed at least 80% of the planned chemotherapy treatments and 93% completed the planned radiation treatments. This compares favorably to previous experience of CRT using similar chemotherapy regimens in other malignancies.

Skin toxicity was an important end-point to evaluate because one of the primary goals in treating many of these patients was to maximize the radiation dose to the skin surface in order to effectively treat the tumor. This issue is quite different when irradiating mucosal carcinomas of the head and neck (e.g., oropharynx, larynx) where minimizing radiation dose to the skin is desired. The added skin toxicity of combining chemotherapy in the setting of head and neck NMSC, is therefore, not known.

Our data suggest that the use of concomitant chemotherapy in the setting of non-skin-sparing radiation treatment in the head and neck is feasible with acceptable skin toxicity since radiation treatment breaks from excessive skin toxicity were rare. In our series, the rate of severe radiation dermatitis was only 13%, which is somewhat surprising since we expected higher rates when tissue-equivalent bolus was used in the majority (75%) of patients. It is likely that the toxicity rates may have been underestimated due to the retrospective nature of clinical record reviews. Clinicians may have been reporting only acute skin toxicity outside the target volume regions. In addition, clinicians may have had difficulty in assessing in-field radiation dermatitis in these patients. Because the treated tumors frequently encompass significant portions of the radiation treatment fields, it may have been difficult to differentiate between in-field dermatitis and tumor necrosis.

Severe mucositis was rare (1 of 15 patients), which was expected in our patients because the majority of the radiation treatment volumes were focused on the skin or unilateral necks, thus, minimizing dose to the oral cavity and pharynx. Hematologic toxicity was also acceptable, primarily due to the use of lower chemotherapy doses in a weekly schedule in the majority of patients.

Due to the small numbers of patients in our study, conclusions cannot be drawn regarding the efficacy of this treatment approach in head and neck NMSCs. And due to the heterogeneity of patient characteristics between patients treated with CRT and RT alone, the therapeutic gain of the addition of chemotherapy to RT cannot be fully assessed in this study. Locoregional control rates with CRT, however, are encouraging, particularly given the high proportion of high-risk features in this patient group as shown in Table 1.

Our results in both the CRT- and RT-treated patients compare favorably with those reported in published literature. Institutional reviews of locally advanced T4 lesions treated with RT alone consistently showed initial local control rates from 50% to 75% for primary lesions [3, 11, 12, 14] and 41% to 50% for recurrent lesions [3, 11]. Ultimate local control rates after salvage therapy in these studies range from 59% to 90% [11, 14] but were frequently associated with significant morbidity. The locoregional control rate in our study was 70% for patients with T4 lesions treated with definitive CRT.

The main limitations of this study are the retrospective nature and small numbers of patients. However, to our knowledge, no other data other than case reports [26, 27] exist on the use of CRT for NMSC of the head and neck. Our experience provides initial data to support additional exploration of this treatment approach for high-risk head and neck NMSCs. Phase I and II studies combining RT with chemotherapy or targeted agents are appropriate to consider in these patients. Based on our institutional experience, a weekly cisplatin or carboplatin regimen would be reasonable to consider as concurrent chemotherapy platforms in future clinical trials, given the acceptable toxicity and ease of administration of these agents. The Trans-Tasman Radiation Oncology Group (TROG 05.01) is currently enrolling patients in a large phase III trial that randomizes resected high-risk (node positive or T3-4) cutaneous squamous cell carcinomas of the head and neck to adjuvant RT with or without concomitant weekly carboplatin. Results from this trial should provide important evidence on the benefit or lack of benefit of adding chemotherapy to RT in these patients. Antiepidermal growth factor (EGFR) agents such as cetuximab or erlotinib would be another approach to consider as radiosensitizing agents, because preliminary reports have suggested that the EGFR pathway may play an important role in the normal physiology of the cutaneous epidermis, [15] and potentially in NMSC metastatic disease [34, 35]. 

This study provides preliminary, hypothesis-generating data on the feasibility and tolerability of combining chemotherapy concomitantly with RT for the treatment of high-risk NMSCs of the head and neck. Our data also confirm previous reports that some patients with NMSC are at high-risk of death and recurrence for their disease. In the absence of proven efficacy, this type of multimodality treatment should at least be considered as an alternative aggressive treatment approach for locally advanced disease or tumors with nodal metastasis. At our institution, we generally consider utilizing concomitant CRT in patients that have unresectable disease (which often include patients with bone invasion) or those with positive margins or nodal disease after surgery. Before becoming a widely accepted treatment approach in these patients, further studies are needed to fully assess the toxicity and better define subsets of patients that may benefit from combined modality treatment.

## Figures and Tables

**Table 1:** Patient and primary tumor characteristics.

	No. (%)	
	CRT	RT
Total number	15	30

Mean age (yrs)	65 (range, 47–84)	66 (range, 48–89)

Gender		
Male	14	26
Female	1	4

Histology		
Squamous cell	11	19
Basal cell	4	11

Location		
Cheek	3	2
Ear	2	5
Eye	1	4
Forehead	2	4
Neck	1	3
Nose	2	1
Preauricular	2	7
Scalp	2	4

T stage		
T1	0	1
T2	0	4
T3	1	2
T4	14	14
Tx	0	9

N stage		
N0	9	22
N1	6	8

Presentation		
Primary	8	11
Recurrent	7	19

Primary treatment		
Definitive	10	10
Postoperative	5	20

Tumor features		
Unresectable	9 (60)	7 (23)
Bone invasion	8 (53)	6 (20)
Nerve invasion	3 (20)	8 (27)
Soft tissue invasion	7 (47)	8 (27)
Positive margins	2 (40)*	9 (45)*
ECE	2 (13)	2 (7)
Multiple recurrence	3 (20)	1 (3)
High grade^†^	9 (90)^††^	8 (62)^††^

*Postoperative patients, ^†^moderately to poorly differentiated, ^††^available tumor grade. CRT, chemoradiotherapy; RT, radiotherapy alone; ECE, extracapsular extension.

**Table 2:** Overall toxicity in chemoradiotherapy patients.

Toxicity	Grade			Total No.
	1	2	3	
Dermatitis	3	10	2	15
Mucositis	0	4	1	5
Conjunctivitis	2	1	0	3
Keratitis	1	0	2	3
Xerostomia	3	1	0	4
Altered mental status	0	0	1	1
Hearing loss	0	1	0	1
Tinnitus	0	1	0	1
Nausea	4	1	0	5
Vomiting	1	1	0	2
Dysphagia	1	0	0	1
Odynophagia	2	0	0	2
Dysgeusia	1	3	0	4
Anorexia	0	2	0	2
Weight loss	0	4	0	4
Fatigue	5	2	0	7
Creatinine elevation	1	0	0	1
Hypocalcemia	1	1	0	2
Hypomagnesemia	4	1	0	5
Hyponatremia	1	0	0	1
Anemia	3	0	0	3
Leukopenia	1	2	2	5
Lymphopenia	2	2	4	8
Neutropenia	1	3	0	4
Thrombocytopenia	3	0	0	3

**Table 3:** Univariate and multivariate hazard ratios of relapse free survival.

Univariate			Multivariate		
Factor	HR (95% CI)	*P* value	Factor	HR (95% CI)	*P* value
Basal cell histology	0.61 (0.3–1.3)	0.40	Bone invasion	9.43 (2.8–32)	<0.01
Well-differentiated	0.93 (0.4–2.2)	0.93	Radiation alone	2.72 (7.9–9.3)	0.11
T4 disease	0.83 (0.4–1.7)	0.72	Nodal disease	2.60 (1.3–5.1)	0.01
Unresectable	0.84	0.76			
Local invasion	2.25 (1.0–4.8)	0.17			
Bone	5.25 (2.5–11.1)	<0.01			
Nerves	1.91 (0.9–4.1)	0.27			
Soft tissue	1.21 (0.6–2.5)	0.73			
Nodal disease	1.35 (0.8–2.2)	0.19			
Positive margins	1.68 (0.8–3.5)	0.34			
Recurrence	1.01 (0.5–2.1)	0.99			
Radiation alone	0.71 (0.3–1.5)	0.53			
Radiation break	2.04 (0.9–4.6)	0.27			
Chemotherapy missed*	3.52 (1.4–8.8)	0.13			

*Applicable to chemoradiotherapy patients. HR, hazard ratio; CI, confidence interval.

**Table 4:** Patterns of failure in chemoradiotherapy patients.

Patient	primary site	T stage	N status	Histology	Adverse features	Type of failure	Time to relapse (months)	Disease status
1	Neck	T4	N0	BCC	Aggressive local invasion	Local	24	NED

2	Nose	T4	N0	SCC	Gross perineural invasion, positive margins	Local	7	DOD

3	Cheek	T4	N0	SCC	Unresectable	Local	6	DOD

4	Cheek	T4	N1	SCC	Unresectable, nodal involvement	Local + regional	5	DOD

5	Scalp	T3	N3	SCC	Nodal involvement	Distant	5	DOD

6	Ear	T4	N2b	SCC	Unresectable, nodal involvement	Distant	9	DOD

Abbreviations: BCC: basal cell carcinoma; SCC: squamous cell carcinoma; NED: no evidence of disease; DOD: died of disease.
